# How different text display patterns affect cybersickness in augmented reality

**DOI:** 10.1038/s41598-024-62338-y

**Published:** 2024-05-22

**Authors:** Jianing Zhang, Xiaoping Che, Enyao Chang, Chenxin Qu, Xiaofei Di, Haiming Liu, Jingxin Su

**Affiliations:** School of Software Engineering, Beijing Jiaotong University, Beijing, China

**Keywords:** Augmented reality, Cybersickness, AR reading, Computer science, Software

## Abstract

Cybersickness remains a pivotal factor that impacts user experience in Augmented Reality (AR). Research probing into the relationship between AR reading tasks and cybersickness, particularly focusing on text display patterns and user characteristics, has been scant. Moreover, the influence of cybersickness on searching ability and the broader spectrum of user experience has not been rigorously tested. Recent investigations have aimed to pinpoint the variables that contribute to cybersickness during AR reading sessions. In one such study, 40 participants underwent a series of controlled experiments with randomized text display patterns, including variations in text speed and text movement modes. Post-experiment, participants completed a questionnaire that helped quantify their experiences and the degree of cybersickness encountered. The data highlighted that satiety, text speed, and text movement mode are significant contributors to cybersickness. When participants experienced higher levels of cybersickness, font color stood out as a particularly influential factor, whereas gender differences seemed to affect the onset of cybersickness more noticeably at lower levels. This study also drew attention to the impact of cybersickness on search ability within AR environments. It was noted that as cybersickness intensity increased, search ability was markedly compromised. In sum, the research underscores the importance of text display patterns and user characteristics, such as past AR experience, in understanding cybersickness and its detrimental effects on user experience and search ability, particularly under conditions of intense cybersickness.

## Introduction

AR technology, while tracing its roots back to the 1990s, didn’t hit the mainstream due to early challenges and a lack of fitting applications. However, the 2020s saw a shift. The advancement of autonomous driving technology, coupled with longer commute times—with Beijing’s average in 2021 hitting 51 minutes—opened up new avenues for AR. This spare time in transit now had the potential to be transformed into productive or leisurely periods, where activities like reading and searching through AR could thrive, thanks to its immersive nature and the ability to overlay digital content onto the real world.

Yet, as the adoption of AR grows within these new spheres, so do the challenges, with cybersickness being a significant hurdle^1^. This discomfort arises from sensory mismatches, where the visual stimuli from the AR interface clash with the physical sensations from the environment, such as the movement of an autonomous car. Such conflicts can lead to symptoms of cybersickness, which, while typically less intense in AR compared to Virtual Reality (VR)^2^, can still manifest as issues with oculomotor function, vestibular reactions, and in extreme cases, nausea. These symptoms can linger until the AR interaction ceases, casting a shadow over the user experience and potentially deterring future use of AR technology.

To improve user interaction with computers, extensive research has been devoted to identifying factors that contribute to cybersickness, with a significant focus on the realm of VR. These studies have examined elements such as VR hardware^3^, the role of music^4^, field of view (FOV)^5^, scene dynamics^6^, scene rotation^7^, and various human factors^8^. Chang E et al. have distilled the causes of cybersickness into three primary categories: hardware, content, and human factors^9^. Understanding these factors is vital for crafting AR experiences and hardware, particularly for productivity-enhancing applications like shop inspections, equipment maintenance, personnel training, military exercises, and simulation drills^10,11^.

Building on these insights, this paper highlights the research voids in AR-related cybersickness, focusing on the underexplored areas of text display patterns and user characteristics. Text display patterns are scrutinized for their effects on reading speed and content searching ability, encompassing various text movement modes (such as horizontal, vertical, or diagonal movements) and the harmony between font and background colors. User characteristics include variables like past AR experience, satiety, and gender. Thus, we propose two critical questions:

*RQ1* Will different text display patterns and user characteristics lead to cybersickness in AR reading?

*RQ2* Is there any correlation between intensity of cybersickness and searching ability in AR reading?

The rest of this paper is organized as follows: In the Sect. ”Related work”, this paper will introduce recent research related to the influencing factors of cybersickness. The third part of this paper will introduce the design of the self- built augmented reality scene and questionnaire in detail. The fourth part analyzes the affect of text display pattern and user characteristics on users’ cybersickness. Subsequently, this article will be discussed in the fifth part and concluded in the sixth part.

## Related work

Cybersickness often manifests through symptoms such as Vestibula, Nausea, Tension and Oculomotor symptoms, all of which can significantly detract from the user experience. This issue represents a substantial barrier to the widespread adoption and advancement of AR technology. Researchers have been investigating the factors that contribute to cybersickness since the 20th century. Before embarking on our study, we conducted a thorough review and assimilation of the latest scholarly articles on the topic. In a pivotal 2020 study, Chang E et al. identified three principal factors contributing to cybersickness: hardware factors, human factors, and content factors^9^.

### Hardware factors

Hardware factors encompass the configuration of AR devices, response time to user input, display technology, and visual fidelity, among others. For instance, Kim K et al. examined how different virtual environment platforms, such as desktop systems, head-mounted displays (HMD), and fully immersive setups, affect emotional engagement and task performance. Their findings suggest that increased levels of immersion correlate with a heightened sense of presence and reality; however, HMDs were found to be the most significant contributors to cybersickness^12^.

### Human factors

Human factors encompass individual differences that can influence susceptibility to cybersickness^6^. Surprisingly, these factors can sometimes be counterintuitive. For example, research by Stoffregen et al. revealed that drivers, rather than non-drivers, are more susceptible to cybersickness during virtual driving simulations, and this likelihood is unaffected by the extent of their real-world driving experience^13^. On another note, Panagiotis et al. investigated how cybersickness impacts cognitive functions and its association with variables such as music, gender, and gaming experience. The findings indicate that subdued music may increase the likelihood of cybersickness, while calming and pleasant tunes can mitigate nausea and overall discomfort associated with cybersickness. Interestingly, gaming experience plays a significant role; individuals with more exposure to video games are less prone to cybersickness. The study also observed that men typically have more gaming experience, which may contribute to gender disparities in cybersickness incidents. Moreover, the research highlighted that cybersickness could considerably impair verbal memory but seems to have a minimal effect on spatial memory capabilities^2^. UMER et al.’s research further supports the notion that gender influences the intensity of cybersickness, suggesting that women, who may be more attuned to their physical sensations, experience more severe symptoms. The study also noted a heightened occurrence and severity of cybersickness symptoms in stressful environments as opposed to more pleasant settings^14^.

### Content factors

Content factors include the effects stemming from alterations in the graphics or task characteristics (such as duration and controllability) within AR environments^9^. Historically, Guna et al.’s research confirmed that content type is a critical factor, with more dynamically intense content increasing the likelihood of cybersickness^15^. Similarly, Saredakis D et al. found that the nature of virtual reality content significantly impacts cybersickness, with 360-degree videos and game content typically scoring higher on the Simulator Sickness Questionnaire (SSQ) than minimalist and scenic content^16^.Katharina et al. demonstrated that incorporating visual and auditory vector cues in XR scenarios can mitigate cybersickness. While a single visual vector cue can alleviate symptoms, an auditory cue alone does not. However, combining auditory with visual cues further reduces the incidence of cybersickness, suggesting that layered vector cues could be an effective mitigation strategy^17^. Wang et al. highlighted frame rate as another contributing factor, pinpointing 120 frames per second as a critical threshold. Cybersickness is more likely when the frame rate drops below this level; maintaining a frame rate above 120 frames per second enhances visual clarity and reaction time, subsequently decreasing the likelihood of cybersickness^6^.

### Summary of related work

This paper contends that while using AR devices for reading and search-related tasks, users are primarily exposed to changes in text display patterns and their own user characteristics. The most common movements during user interaction are along single axes, including vertical and horizontal shifts, as well as depth-based adjustments. Sometimes, there is also engagement with dual-axis movements, such as those along the yz and xy planes. The velocity at which these movements occur further influences the user experience. Considering the paucity of research on the effect of these specific motion modes on cybersickness, this paper advocates for a more comprehensive examination of this subject in future studies.

Additionally, as AR devices are increasingly employed in real-world environments, the significance of background color in reading and search tasks cannot be overlooked^18^. When the text to be read or located is similar in color to the background, it may require more effort from users to distinguish it, potentially heightening the risk of cybersickness. The relationship between font color consistency and background color in AR has not been extensively studied, indicating a gap in the literature. Therefore, this paper suggests that future research should explore the impact of font and background color consistency on cybersickness for a more in-depth understanding of how to enhance user experience with AR technology.

**Figure 1:**
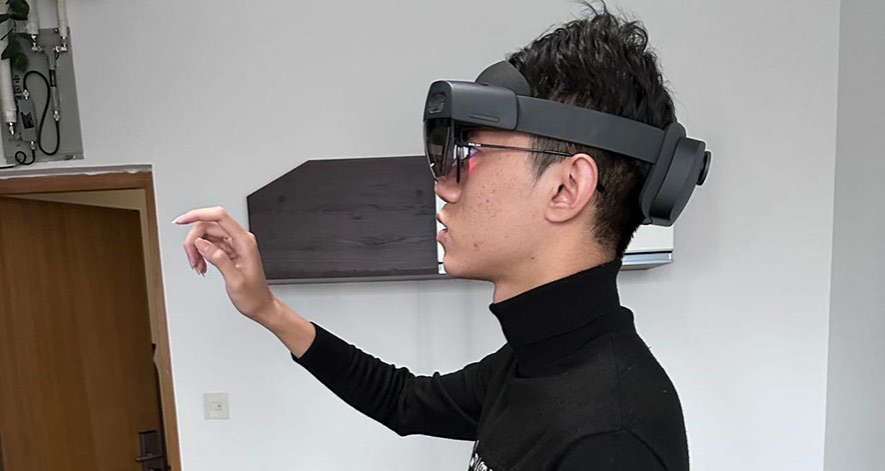
Experimenters are conducting experiments in different text movement modes with Microsoft Hololens 2.

In conclusion, our review has identified a gap in the field of AR cybersickness research, specifically regarding the effects of text movement mode consistency and the harmony of font color with background color. To address this, our paper intends to first examine the influence of font color on cybersickness. Subsequent studies will aim to investigate the relationship between font and background color consistency and their collective impact on cybersickness in AR environments. We also acknowledge the importance of subjective factors and, therefore, plan to delve into the role of human factors within these contexts (Fig. 1).

## AR environment and questionnaire design

### AR environment design

Our study was designed with meticulous care, setting up six unique scenarios that included a control group and five experimental conditions, details of which are outlined in the subsequent sections. To control for external variables, we streamlined the experiment to include only the essential elements necessary for the reading tasks. In the control group, text was held static, using the default white font color provided by Unity. Each experimental condition consisted of a three-part structure: participants engaged in two search tasks and a reading task. To clearly signify the task at hand, each scenario was prominently marked with a “Reading Test” sign above. We utilized C# for object motion control and Unity for scene construction, with the final deployment carried out on the Microsoft HoloLens 2.

### Text display pattern

In our experiment, we meticulously categorize text display patterns into three distinct segments for analysis: color, speed, and text movement modes.

**Figure 2:**
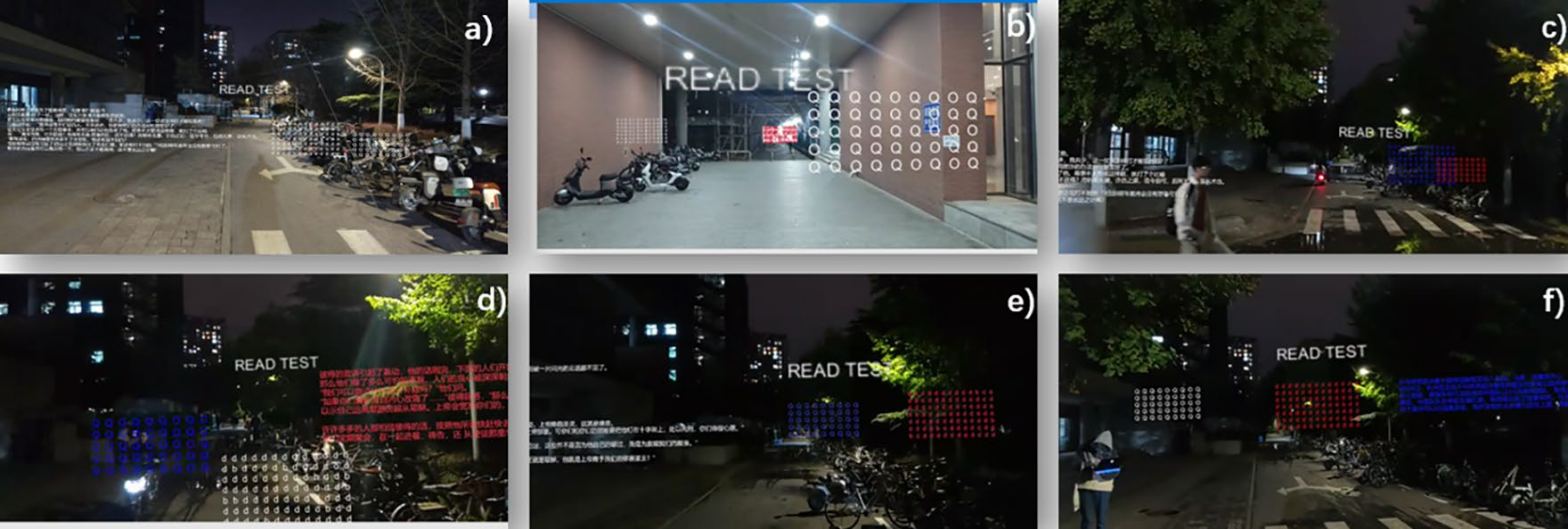
Experimental Scenarios. (**a**) Experiment 0: Control group; (**b**) Experiment 1: Rolling scene; (**c**) Experiment 2, X-axis oscillation; (**d**) Experiment 3, Z-axis oscillation; (**e**) Experiment 4, Y-axis oscillation; (**f**) Experiment 5, Dual-axis oscillation.

**Font color** The aspect of color in our study is explored through the lens of font color selection. This facet is intricately woven into our examination of text movement modes. Drawing from our comprehensive literature review^19,20^, we understand that color temperature—classified as warm or cool—affects the reading experience on mobile devices. To assess if this impact is mirrored in AR environments, we present a spectrum of colors including a cool **blue (RGB(0,0,255))**, a neutral **white (RGB(255,255,255))**, and a warm **red (RGB(255,0,0))**, as shown in Fig. 2. We use blue to represent cool colors and red to represent warm colors.

Further, our initial findings recognize that the contrast between font color and background is not just a matter of visual appeal but is a critical component in enhancing readability. This dynamic interplay between font and background color deserves a deeper dive in our future research for a more intricate understanding of its role in the user reading experience.

**Text movement mode** Our study rigorously designed an array of six text motion scenarios within a confined 5-meter space to ensure the experimental space remained manageable for observation,, focusing on their influence on AR reading comprehension. We delineate the motion scenarios as follows:

**Control group** The text is static, providing a reference point for comparative analysis. **Rolling Scene:** Text vertically scrolls in a designated loop, replicating the dynamic flow of information. **X-axis Oscillation:** Text engages in a horizontal, to-and-fro motion, oscillating along the X-axis. **Z-axis Oscillation:** Text executes a reciprocating motion along the Z-axis, moving closer and further from the reader. **Y-axis Oscillation:** Text bobs up and down, creating an oscillatory movement along the Y-axis. **Dual-axis Oscillation:** Text moves in a complex, reciprocating pattern along a diagonal trajectory that intersects the Y-axis and Z-axis. These scenarios have been specifically created to replicate a variety of text movement patterns, which allows us to evaluate the impact on cybersickness, user experience, and search proficiency across different text movement modes. With this methodology, we aim to gain a comprehensive understanding of how text movement modes influence reading engagement in an AR setting. These Text Movement Modes are displayed in Fig. 2.

Given that the scenarios we are simulating pertain to AR reading, we have carefully selected several text movement patterns that users most commonly encounter during the AR reading process to serve as the foundation for our experiments.

**Text speed**: In our experiments, we meticulously calibrated text movement speeds to a tiered system, ensuring legibility and detectable motion. We set the high speed at 2.5 meters per second, the moderate speed at 2 meters per second, and the low speed at 1 meter per second. The three speed manipulations we selected were based on a small-scale preliminary survey involving 8 participants. In this survey, we set up 10 different speed settings for scenarios involving X-axis oscillation: .5 m/s, 1 m/s, 1.5 m/s, 2 m/s, 2.5 m/s, 3 m/s, 3.5 m/s, 4 m/s, 4.5 m/s, and 5 m/s. After collecting feedback from the participants, we found that 6 of them reported almost being unable to clearly see the text when the speed exceeded 2.5m/s, while a speed of .5 m/s was too slow, making it feel almost stationary. Additionally, 4 participants indicated that 2 m/s felt the most comfortable. Based on this feedback, we ultimately selected 1m/s, 2m/s, and 2.5 m/s as our three speed manipulations.This tiered approach allows us to scrutinize the effects of different speeds on the AR reading experience without compromising text clarity.

The search tasks were devised to test visual sharpness and attention to detail. Participants were tasked with finding several concealed ’d’ within a field of 91 ’b’ characters, arranged in a 13x7 grid, and locating several hidden ’O’ amongst 45 ’Q’ characters in a 9x5 grid, as shown in Fig. 1.

**Figure 3:**
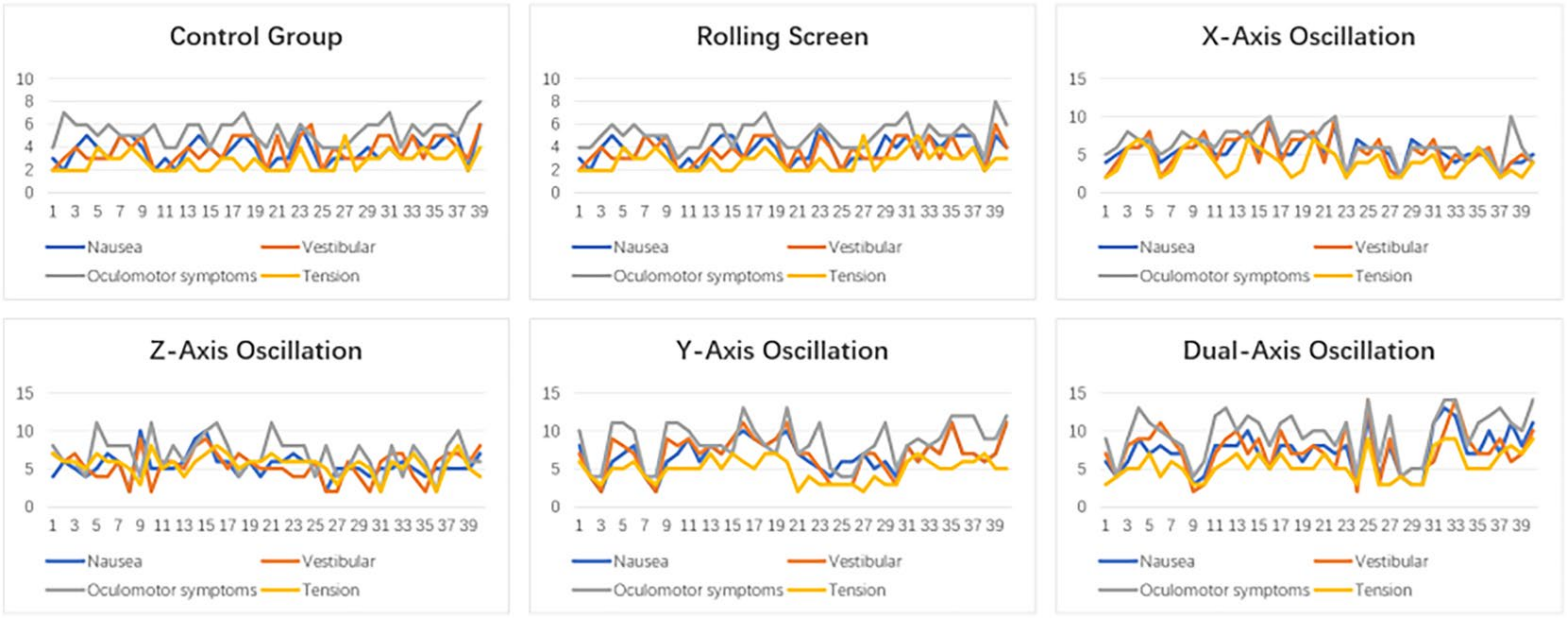
Experimental data line graph.

In selecting the reading material, our preliminary research indicated that participants possessed a degree of familiarity with biblical narratives, idiomatic expressions, and fairy tales. Consequently, we incorporated these stories as the substrate for our reading comprehension tests. Following the experiment, we engaged with participants through semi-structured interviews to probe their comprehension of the material and to explore the potential impact of cybersickness on their reading retention (Fig. 3).

### Questionnaire design

This study presents an enhancement of the “Cybersickness in Virtual Reality Questionnaire” (CSQ-VR) to broaden its utility for assessing cybersickness in AR settings. The CSQ-VR, originally developed based on symptoms triggered by virtual reality experiences and grounded in neuroscientific questionnaire frameworks, has shown outstanding validity^21^. Research corroborates that CSQ-VR exceeds the reliability of traditional cybersickness surveys such as the Simulator Sickness Questionnaire (SSQ) and Virtual Reality Sickness Questionnaire (VRSQ)^22^.

The streamlined CSQ-VR encompasses six items, vastly increasing its succinctness and clarity for participants^22,23^. It evaluates the full spectrum of cybersickness symptoms: nausea (incorporating feelings of nausea and dizziness), vestibular disturbances (including disorientation and balance issues), oculomotor disturbances (covering visual strain and eye discomfort), and general discomfort (such as sweating and excessive salivation). Each category is represented by two targeted questions, formatted on a seven-point Likert scale ranging from “1—No sensation” to “7—Intense sensation,” with responses incorporating both descriptive language and numerical values. The questionnaire calculates an aggregate score along with subscores for each symptom type—nausea, vestibular, oculomotor, and general discomfort. Subscores are determined by totaling the points for relevant questions, while the aggregate score sums up these subscores.

In addition, we designed a questionnaire to assess user’s reading, searching capabilities in AR and collect their characteristics. The search ability score is a composite of points from Q1 and Q2, where a misstep in Q1 incurs 2 points and an error in Q2 assigns 1 point, indicating that higher scores reflect a greater detriment of cybersickness on search efficiency. This instrument aims to probe the effects of text motion and font color on reading and search performance in AR contexts. The questionnaire posed are as follows:

**Table 1:** The collection of user information.

User parameter questionnaire	
1. What is your age?	
2. What is your gender?	
A. Male B. Female	
3. How would you describe your experience with AR devices?	
A. Inexperienced (3 times or fewer) B. Experienced (more than 3 times)	
4. Do you feel satiated?	
A.Satiated B.Not satiated	
Q1: How many ‘O’ s are concealed among the ‘Q’ s?	
Q2: How many ‘b’ s are concealed among the ‘d’ s?	
Q3:To what extent do you believe font color impacts your cybersickness?	
(Rate on a scale from 1 to 7, where 1 conveys no impact and 7 signifies a pronounced impact)	
Q4:To what extent do you believe the speed of text movement influences your cybersickness?	
(Rate on a scale from 1 to 7, where 1 denotes no impact and 7 reflects a significant impact)	

### User characteristics, participants and procedures

Our study were approved and endorsed by the School of Software, Beijing Jiaotong University. In this study, we strictly adhered to the ethical standards involving human participants. We informed all participants and/or their legal guardians about the nature, purpose, potential risks, and benefits of the study, and we have explicitly obtained their informed consent. Furthermore, considering that the manuscript may contain identifying information or images, we have obtained specific consent from the participants in Fig. 1, allowing these details and images to be published in an online open-access publication.

For the study, our participant selection was based on a combination of user characteristics: satiety(We controlled for this variable by telling participants whether or not they ate before the experiment. Participants were divided into two categories: those who had dinner were considered satiated, and those who did not were considered not satiated.), gender, and past AR experience. We screened and recruited a total of 40 participants (All participants confirmed the absence of language barriers, and all possess normal vision without any symptoms of color blindness.), primarily college or graduate students aged between 18 and 25 (M = 20.5, SD = 3.16), with an equal gender split of 20 males and 20 females. Within each gender group, the breakdown was as follows: 5 satiated individuals with past AR experience, 5 unsatiated with past AR experience, 5 satiated without past AR experience, and 5 unsatiated without past AR experience.

Choosing to conduct our experiments on streets during dusk was a deliberate move to sidestep the intense glare of midday sunlight, which could potentially interfere with the AR apparatus. We leveraged the soft lighting from street lamps to create optimal conditions for our experimental tasks.

Before commencing the experiment, we employed GPT-4.0 to generate individualized experimental sequences for each participant to introduce task variability and minimize the risk of cybersickness due to prolonged exposure rather than the experimental conditions. Once the experiment was underway, we initiated a timer, and participants began navigating through the six experimental phases (Control Group, Rolling Scene, X-axis Oscillation, Z-axis Oscillation, Y-axis Oscillation, and Dual-axis Oscillation) in accordance with their unique sequence numbers. After each phase, they filled out corresponding questionnaires.

Upon completion of all phases, we conducted semi-structured interviews to collect in-depth qualitative insights into the participants’ experiences and subjective reactions to the experiment.

Should any participant encounter intense cybersickness, leading to an inability to continue, we paused the timer and recorded the time elapsed. The session was halted to allow time for recovery. We resumed the remaining experimental tasks only after the participant had fully recuperated from any cybersickness symptoms. To evaluate the potential correlation between cybersickness and the duration of task involvement, we then administered an additional Control Group session that mirrored the previously recorded duration.

### Hypotheses and exploratory aims

Based on our research question, investigation and literature review, we propose the following assumptions:

#### H1

Cybersickness is mainly reflected in Oculomotor symptoms.

#### H2

The influence of speed is more significant than color.

#### H3

Cybersickness will affect the search ability of participants and reduce user experience.

#### H4

Female participants and experimenters without Past AR Experience will experience a stronger cybersickness^24^.

#### H5

Satiety experimenters will experience a stronger cybersickness.

This article extends its inquiry beyond preliminary assumptions to explore the interplay between Past AR Experience and the four fundamental symptoms of cybersickness. While prevailing studies have investigated how cybersickness can affect reading proficiency, our research shifts focus towards understanding its influence on search capabilities. Furthermore, we will examine additional demographic information to discern potential correlations or impacts on cybersickness, aiming to uncover the reasons behind the contradictory reports on gender differences in cybersickness found in the existing literature.

## Measures

We quantified the data collected in Table 1 using Table 2. Participants without AR experience were assigned 1 point, while those with AR experience received 0 points. Participants who were satiated received 1 point, and those who were not satiated received 0 points. An incorrect answer to Q1 was given 2 points, while an incorrect answer to Q2 received 1 point. Additionally, the scores for Q3 and Q4 were added to the CSQ-AR score to determine the participant’s subjective impact of speed/color on cybersickness.

**Table 2:** The collection of user information.

Data type	Data classification	Data quantization
AR Experience	Inexperienced (3 times or fewer)	1
	Experienced (more than 3 times)	0
Satiety	Satiated	1
	Not Satiated	0
Number of O	Wrong	2
	Right	0
Number of b	Wrong	1
	Right	0

## Results

The statistical analysis for this study was conducted using SPSS^25^, while the creation of the violin charts was achieved through Raincloud-shiny. To determine the consistency of our data, Levene’s test for homogeneity of variances was applied. The p-values for both color and speed within the six AR scenes were above .05, suggesting that the variances for each factor are homogenous. We then utilized a Two-Way ANOVA to examine the effects of text speed and font color on searching ability and the overall cybersickness, as well as on specific symptoms such as Nausea, Vestibular, Oculomotor symptoms, and Tension^26^. To explore the impact of Nausea, Vestibular, Oculomotor Symptoms, Tension, and overall cybersickness on user experience and searching ability, we applied Mixed Regression Model analysis. This method was also used to assess the effects of gender, satiety, and past AR experience on the severity of cybersickness^27^. A T-test was conducted to assess the influence of experimenter satiety and past AR experience on cybersickness^28^. Lastly, an ANCOVA with Satiety as a covariate was performed to evaluate whether past AR experience and female participants indeed experience significantly different levels of cybersickness^29^.

### Cybersickness and text display pattern

We will examine text display patterns from the perspectives of Text Movement Mode, Speed, and Color.

Different text movement modes result in varying levels of cybersickness across experimental groups. We assess the intensity of cybersickness for each group by using the average halo score from questionnaires completed by the participants. The halo score is derived by summing the total scores for Nausea, Vestibular, Oculomotor Symptoms, and Tension. Table 3 displays these findings, with Experiment 0 acting as the Control Group, indicating the level of cybersickness experienced with static AR text display.

**Table 3:** Mean Value of overall Cybersickness (Cyber), Nausea(Nau), Vestibular (Vest), Oculomotor symptoms (Ocu) and Tension (Ten).

Test	Cyber	Nau	Vest	Ocu	Ten
Dual-axis oscillation	29.98	7.35	7.45	9.70	5.48
Y-axis oscillation	27.13	6.83	6.90	8.55	4.85
Z-axis oscillation	22.25	5.45	5.58	7.08	4.15
X-axis oscillation	20.65	5.23	5.15	6.33	3.95
Rolling scene	15.25	3.75	3.75	5.00	2.75
control group	15.4	3.7	3.25	5.2	2.75

Based on the measured intensities of cybersickness, we can categorize them into five distinct levels: very weak, weak, medium, strong, and very strong. Notably, Oculomotor symptoms seem to be the most significant contributor to the overall cybersickness score in each experimental condition. These observations align with our initial hypothesis, H1.

**Figure 4:**
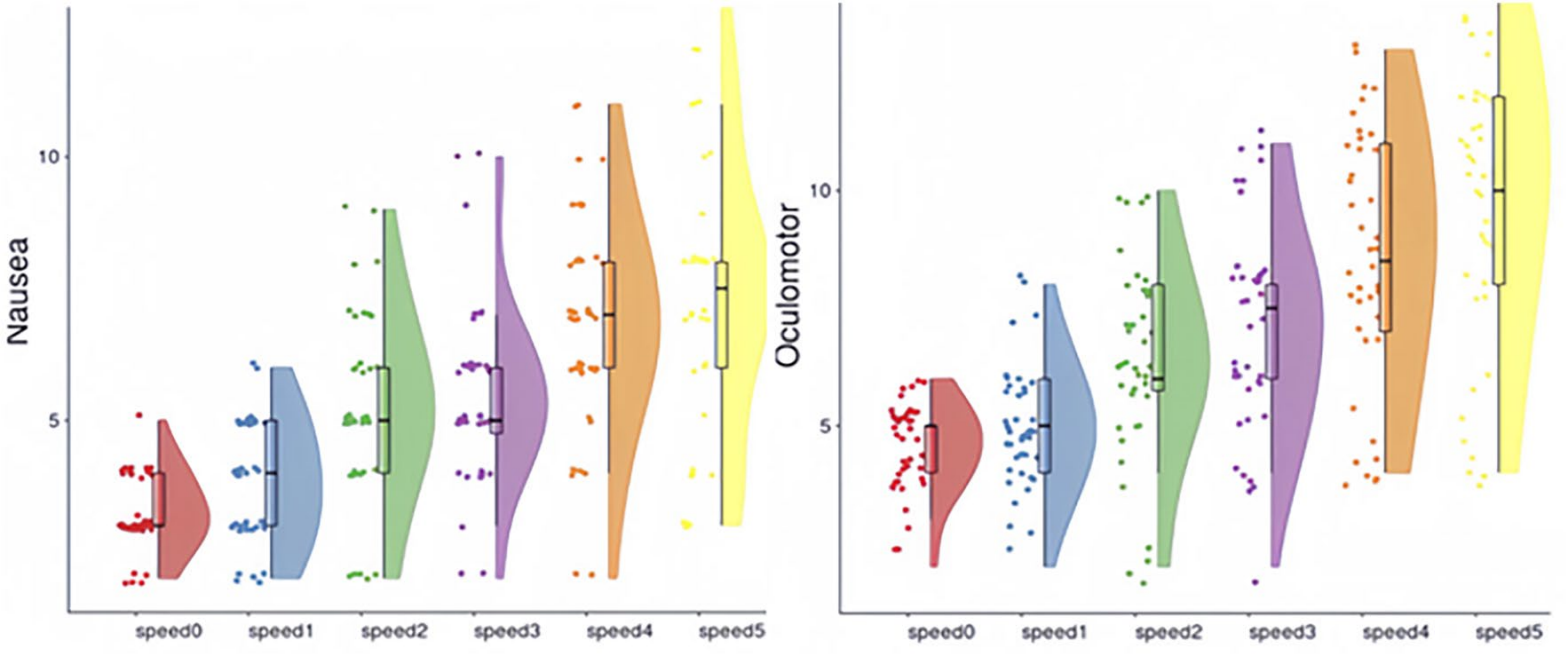
Nausea (Left) and oculomotor (right) intensities by speed in each text display pattern.

**Figure 5:**
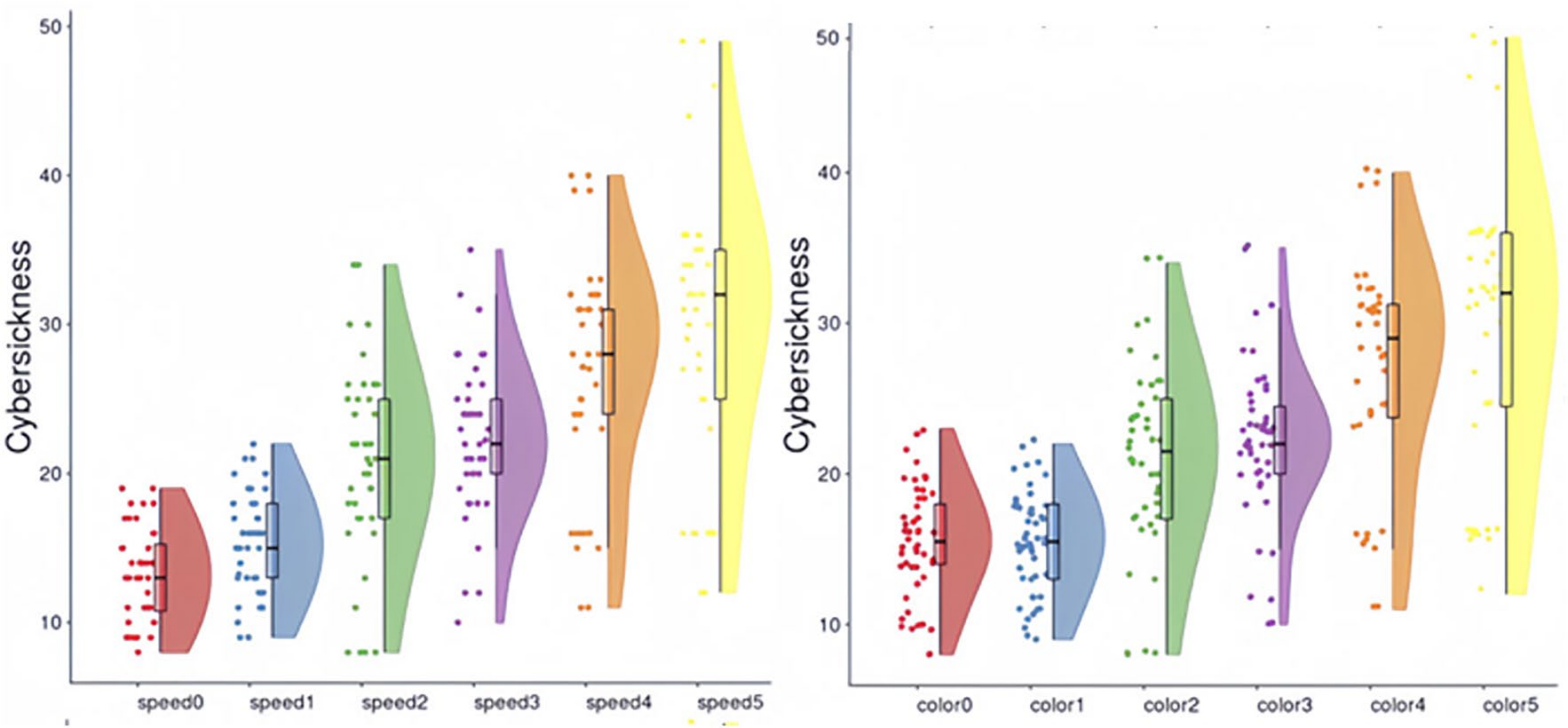
Overall cybersickness (left) and by speed in each text display pattern. overall cybersickness(right) intensities by color in each text display pattern.

Conversely, a Two-way ANOVA revealed no significant interaction between speed and color in relation to cybersickness. Nevertheless, in the five experimental groups (numbered 1 to 5), the influence of speed on Nausea, Vestibular, Oculomotor symptoms, Tension, and overall cybersickness is documented in Table 4. Due to the similarity of the cybersickness scores in the Rolling scene to those of the Control Group, suggesting minimal intensity insufficient to trigger cybersickness, this condition was excluded from the analysis. Our findings indicate that speed significantly affects Nausea and Oculomotor symptoms, as illustrated in Figs. 4 and 5. Notably, within the Y-axis oscillation condition, speed’s impact was found to be most pronounced across all measured aspects.

**Table 4:** Two way ANOVA for speed and color in nausea, vestibular, oculomotor, tension and overall cybersickness.

Test	Predicted	Predictor	F	*p*-value	$\omega ^2$
Rolling scene	Nausea	Speed	.79	.39	.01
		Color	1.76	.19	.03
	Vestibular	Speed	.20	.66	− .03
		Color	.97	.22	.01
	Oculomotor	Speed	.56	.42	− .07
		Color	.98	.34	.01
	Tension	Speed	.074	.96	− .02
		Color	.54	.47	.02
	Cybersickness	Speed	.69	.41	.00
		Color	1.25	.27	.02
X− axis oscillation	Nausea	Speed	8.52	<.01	.18
		Color	.33	.57	.00
	Vestibular	Speed	7.15	<.05	.13
		Color	1.21	.14	.03
	Oculomotor	Speed	1.14	.29	.01
		Color	1.24	.27	.02
	Tension	Speed	5.63	<.05	.14
		Color	5.63	<.05	.04
	Cybersickness	Speed	13.01	<.001	.25
		Color	.69	.41	.01
Z-axis oscillation	Nausea	Speed	5.09	<.05	.12
		Color	.13	.72	− .03
	Vestibular	Speed	1.02	.32	.02
		Color	1.96	.17	.04
	Oculomotor	Speed	7.66	<.01	.17
		Color	.14	.72	− .03
	Tension	Speed	.45	<.05	.16
		Color	.16	.69	.00
	Cybersickness	Speed	5.72	<.05	.12
		Color	.77	.39	.01
Y-axis oscillation	Nausea	Speed	10.14	<.005	.22
		Color	.73	.40	.03
	Vestibular	Speed	22.47	<.001	.38
		Color	.20	<.66	.01
	Oculomotor	Speed	28.97	<.001	.41
		Color	2.74	.11	.02
	Tension	Speed	6.89	<.05	.01
		Color	.02	.89	− .03
	Cybersickness	Speed	24.59	<.001	.40
		Color	.16	.69	− .02
Dual-axis oscillation	Nausea	Speed	5.87	<.05	.08
		Color	10.81	<.005	.18
	Vestibular	Speed	3.10	.86	.05
		Color	7.78	<.005	.21
	Oculomotor	Speed	10.26	<.005	.13
		Color	14.33	<.001	.20
	Tension	Speed	4.30	.11	.07
		Color	8.33	<.05	.15
	Cybersickness	Speed	7.36	<.01	.10
		Color	13.33	<.005	.21

The effect of color in the Dual-axis oscillation was so dominant that when used as a covariate, the corrected influence of speed on Nausea [F (1, 37) = 5.87, $$ p<$$.05, $$\omega ^2$$ = .14], Vestibular [F (1, 37) = 3.1, *p* = .085, $$\omega ^2$$ = .077], Oculomotor symptoms [F (1, 37) = 10.26, $$ p<$$.005, $$\omega ^2$$ = .22], Tension [F (1, 37) = 4.30, $$ p<$$ .05, $$\omega ^2$$ = .104], and overall cybersickness [F (1, 37) = 7.36, p < .05, $$\omega ^2$$ = .166] became more apparent, as the adjusted $$r^2$$ values were higher post-correction. This suggests that in the Dual-axis oscillation, the impact of speed on cybersickness was confounded by the presence of color.

Further analysis of the five experimental groups shows that in conditions of extremely strong cybersickness intensities, color significantly influences overall cybersickness, Nausea, Vestibular, Oculomotor symptoms, and Tension, as seen in Fig. 3. Contrary to hypothesis H2, color has a more substantial effect than speed in these situations. However, when cybersickness intensities are low, color does not significantly impact the condition.

### Cybersickness on searching ability and user experience

**Figure 6:**
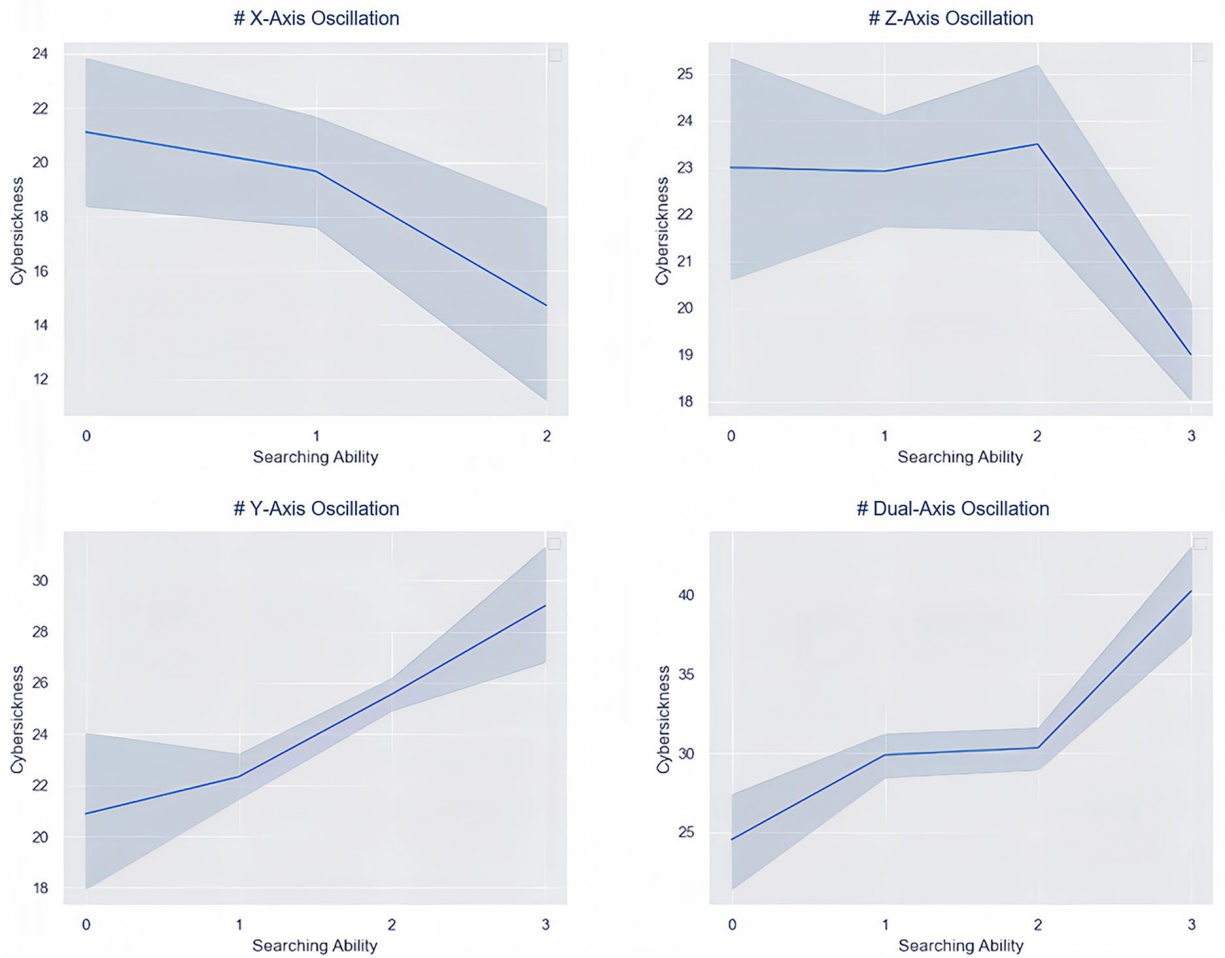
Overall Cybersickness’s Effect on Searching Ability (A higher score indicates a lower searching ability) in Experiment2 (Top Left), Experiment3 (Top Right), Experiment4 (Bottom Left), Experiment5 (Bottom Right).

Cybersickness detrimentally influences both search ability and user experience, supporting hypothesis H3 as indicated in Table 5 and Fig. 6. Cybersickness notably degrades user experience and, under specific circumstances, significantly hampers search ability. The data in the table reveals that search performance was particularly compromised in two experiments where cybersickness levels reached “strong” and “very strong.” When cybersickness is milder, its impact on searching ability is negligible. Moreover, Vestibular and Oculomotor Symptoms are identified as principal contributors to the decline in search ability (refer to Table 5 for t-values and $\beta$ coefficients).

**Table 5:** Mixed regression models for searching ability and user experience.

Predicted	Predictor	F	t	$\beta$	*p*-value	$\omega ^2$
Searching ability-dual-axis oscillation	Nausea	15.88	3.98	.54	<.001	.30
	Vestibular	21.41	4.63	.60	<.001	.36
	Oculomotor	29.75	5.45	.66	<.001	.44
	Tension	6.93	2.63	.39	<05	.15
	Cybersickness	23.91	4.90	.62	<.001	.39
User experience-dual-axis oscillation	Nausea	10.13	3.18	.46	<0.05	.21
	Vestibular	12.06	3.47	.49	<0.05	.24
	Oculomotor	6.47	2.54	.38	<.05	.15
	Tension	7.92	2.82	.42	<.01	.17
	Cybersickness	11.34	3.37	.48	<.005	.23
Searching ability-Y-axis oscillation	Nausea	5.55	2.35	.36	<.05	.13
	Vestibular	4.67	2.16	.33	<.05	.11
	Oculomotor	10.22	3.19	.46	<.005	.21
	Tension	.20	.44	.07	.66	.01
	Cybersickness	5.88	2.42	.37	<.05	.13
User experience-Y-axis oscillation	Nausea	18.93	4.35	.58	<.001	.33
	Vestibular	11.86	3.44	.49	<.005	.24
	Oculomotor	1.67	1.29	.21	.21	.04
	Tension	5.01	2.24	.34	<.05	.12
	Cybersickness	11.17	3.34	.48	<.005	.23
Searching ability-Z-axis oscillation	Nausea	.01	.05	,01	.10	− .03
	Vestibular	.63	− .79	− .13	.43	.02
	Oculomotor	.000	2.31	.001	.99	− .02
	Tension	4.42	− 2.10	− .32	<.05	.10
	Cybersickness	.43	− .66	− .11	.51	.01
User Experience-Z-axis oscillation	Nausea	3.19	1.79	.28	.08	.08
	Vestibular	19.58	4.42	.58	<.001	.34
	Oculomotor	3.52	1.88	.29	.07	.09
	Tension	4.05	2.01	.31	.05	.11
	Cybersickness	11.51	3.39	.48	<.005	.23
Searching Ability-X-axis oscillation	Nausea	.255	.505	.082	.62	.08
	Vestibular	.14	.38	.06	.71	.01
	Oculomotor	.10	.32	.05	.75	.01
	Tension	2.21	1.49	.235	.15	.06
	Cybersickness	.56	.75	.12	.46	.02
User Experience-X-axis oscillation	Nausea	16.76	4.09	.55	<.001	.31
	Vestibular	21.08	4.60	.60	<.001	.36
	Oculomotor	15.40	3.92	.54	<.001	.29
	Tension	4.02	2.00	.31	.052	.11
	Cybersickness	21.39	4.62	.60	<.001	.36
Searching Ability-Rolling scene	Nausea	4.86	2.2	.34	<.05	.11
	Vestibular	2.14	1.46	.23	.15	.053
	Oculomotor	3.60	1.90	.29	.07	.09
	Tension	.47	.68	.11	.50	.01
	Cybersickness	4.46	2.11	.32	<.05	.11
User experience-rolling scene	Nausea	13.08	3.61	.51	<.005	.26
	Vestibular	16.02	4.00	.55	<.001	.30
	Oculomotor	9.39	3.06	.44	<.005	.20
	Tension	s 1.07	1.03	.17	.31	.03
	Cybersickness	16.80	4.10	.55	<.001	.31

Vestibular symptoms consistently and significantly affect user experience regardless of cybersickness intensity. Other symptoms like Nausea also exert a considerable influence on user experience in certain scenarios. However, Tension seems to have a minimal effect on both searching ability and user experience in most instances (see $\beta$ in Table 5). This could suggest that the impact of Tension is perhaps not as pronounced due to its lower intensity. Furthermore, the control group data, with an average search ability score of .1 and a variance of .09, implies that AR device usage alone is unlikely to impair searching ability. The result of this part answers RQ2.

### Cybersickness and user characteristics

According to Table 6, satiety and past AR experience emerge as the sole demographic predictors of cybersickness severity, aligning with hypothesis H5 but veering away from H4. Notably, individuals who have fasted or have past AR experience exhibit significantly lower overall cybersickness levels.

**Table 6:** Mixed regression models for cybersickness: user characteristics (gender, satiety and past AR experience (Past AR)).

Test	Predictor	F	t	$\beta$	*p*-value	$\omega ^2$
Dual-axis oscillation	Gender	.51	.72	.12	.48	.01
	Satiety	12.38	3.52	.50	<.005	.25
	Past Ar	5.23	2.29	.35	<.05	.12
Y-axis oscillation	Gender	.86	− .93	− .15	.36	.02
	Satiety	21.89	4.68	.61	<.001	.37
	Past Ar	10.53	3.25	.47	<.005	.22
Z-axis oscillation	Gender	1.02	− 1.01	− .16	.32	.03
	Satiety	59.27	7.70	.78	<.001	.61
	Past Ar	13.87	3.72	.52	<.005	.27
X-axis oscillation	Gender	14.92	− 3.86	− .53	<.001	.28
	Satiety	7.58	2.75	.41	<.01	.17
	Past Ar	17.51	4.19	.56	<.001	.32
Rolling scene	Gender	4.33	2.08	.32	<.05	.10
	Satiety	22.56	4.75	.61	<.001	.37
	Past Ar	.66	.81	.13	.42	.02

Table 6also reveals that gender significantly predicts the severity of cybersickness only in scenarios involving the Rolling scene and X-axis oscillation where cybersickness intensities are low. Conversely, satiety and past AR experience are robust predictors of cybersickness severity, as evidenced by T-test outcomes. In the Dual-axis oscillation, both the satiety participant group and those lacking past AR experience reported heightened cybersickness symptoms ($p<$.05). The same trend of increased symptoms for the satiety participants and lacking past AR experience individuals was observed across Y-axis and Z-axis oscillations ($p<$.05). In the X-axis oscillation, female participants, full participants, and those without past AR experience were notably more affected by cybersickness. Similarly, in the Rolling scene, female participants experienced more pronounced symptoms.

Further analysis using covariance, with satiety as a covariate in ANCOVA, indicated that past AR experience did not significantly impact cybersickness in the Rolling scene(*p* = .9), which had very low cybersickness intensities. However, gender did have a significant effect on cybersickness in both the Rolling scene and X-axis oscillation ($p<$.05). These findings will be discussed in further detail in the Discussion section. The above studies answer RQ1.

## Discussion

**Text display pattern and cybersickness** In this study, speed emerged as the predominant factor significantly impacting cybersickness. The interplay between speed and the complexity of the text movement modes effect around objects suggests that intricate text movement modes exacerbate the influence of speed on cybersickness. The investigation reveals that speed primarily contributes to cybersickness by exacerbating Nausea and Oculomotor symptoms. As the intensity of cybersickness escalates, the influence of speed on Vestibular symptoms also increases, as indicated by the $R^2$ values in Table 4.

Participant feedback underscores that higher speeds tend to intensify Vestibular symptoms and overall cybersickness. Notably, in the Y-axis oscillation scenario, speed has the most pronounced impact. This finding implies that speed in the Y-axis oscillation’s motion mode—where objects appear to move towards the observer—is a crucial element affecting AR-induced cybersickness. This particular movement mode resembles the experience of forward movement, similar to what passengers feel while in transit.

The onset of cybersickness during reading and searching activities in AR is hypothesized to arise from a sensory mismatch: the user’s eyes, focusing on the moving text displayed by the AR device, signal to the brain that the environment is in motion. However, without corresponding movement detected by the inner ear, a perceptual conflict occurs between visual input and vestibular sensation, leading to cybersickness. This phenomenon is documented in the referenced study^30^.

Color has a significant influence on the overall severity of cybersickness and its associated symptoms—Nausea, Vestibular, Oculomotor, and Tension—particularly during Dual-axis oscillation where cybersickness intensities are very strong. Contrastingly, in scenarios such as the Rolling scene, X-axis, Z-axis, and Y-axis oscillations, color does not appear to have a notable effect on these symptoms. This suggests that color’s impact on cybersickness becomes more prominent as the intensity of cybersickness crosses a certain threshold. Below this level, color’s effect is not significant. This observation aligns with experiences of carsickness, where passengers may feel discomfort due to the glare of oncoming headlights at night.

Drawing from the insights regarding text movement mode and its relation to cybersickness, we can offer practical guidance for AR content developers. To minimize users’ cybersickness during AR-based reading or searching tasks, it is recommended to restrict text motion to a single axis when possible. A comparative analysis of X-axis, Z-axis, and Y-axis oscillations suggests that movements along the y-axis tend to induce more cybersickness than those along the x-axis or z-axis. This implication can be pivotal for designing user-friendly AR applications that prioritize comfort and usability.

To address the issue of poor measurement effectiveness for the “Tension” variable in the CSQ-AR questionnaire, we believe that a combination of subjective and objective data could improve the situation. Specifically, we plan to collect physiological data from participants, such as eye movement, galvanic skin response, and heart rate, to objectively reflect their state of tension. Additionally, during semi-structured interviews, we will ask questions related to the “Tension” to gather participants’ personal feelings and subjective opinions. Moreover, we will continuously monitor participants’ behaviors during the experiment, such as hand tremors, speech rate, and tone changes, which may indirectly indicate their level of tension. Through such multidimensional observation and analysis, we aim to establish a more comprehensive and accurate mechanism for measuring “Tension”.

**Searching ability, user experience and cybersickness** Recent studies have confirmed that cybersickness considerably affects user experience, echoing past research on VR-induced cybersickness. Vestibular symptoms, in particular, have a definitive and increasingly negative impact on User Experience as cybersickness becomes more severe. This mirrors the sensory conflict between the inner ear and vision that is typical in cybersickness. Consequently, strategies that alleviate Vestibular symptoms, such as taking preemptive medication or performing eye exercises, may prove beneficial in improving User Experience. For developers, smoothing out rapid transitions in AR scenarios can also play a role in curbing cybersickness and enhancing the overall experience.

Furthermore, it has been observed that search ability is significantly compromised during Y-axis and Dual-axis oscillations, with Vestibular and Oculomotor symptoms being the primary culprits. It is hypothesized that when cybersickness reaches a certain intensity, it negatively affects the experimenter’s ability to search. This could be due to cybersickness crossing a threshold that hinders cognitive functions, leading to decreased reaction times, diminished attention, and impaired judgment. Such effects are akin to those experienced under the influence of alcohol or lack of sleep, such as the difficulty in quickly and accurately identifying a target letter, and are in line with the research findings of Panagiotis et al in their reading ability research^4^.

**User characteristics in cybersickness** Satiety stands out as a consistent predictor of cybersickness across various levels of intensity, with a notable increase in symptoms when participants are satiated. This could be due to the increased digestive activity after eating, which requires more blood flow and, in turn, could reduce the blood supply to the brain, impacting balance and spatial orientation. Additionally, the activity of the digestive system may interfere with the vestibular system, exacerbating the effects of cybersickness. However, contrasting evidence from carsickness studies indicates that not all individuals experience heightened symptoms post-meal, suggesting that additional factors related to fullness could be at play, marking an intriguing direction for future research.

Gender has also emerged as a significant predictor of cybersickness in scenarios like the Rolling scene and X-axis oscillation, with female participants reporting greater severity. This could be due to a heightened awareness of bodily sensations among women, particularly when cybersickness is mild, which is consistent with the findings of UMER et al.^18^.

While past AR experience is often considered a key factor in cybersickness, it did not significantly predict the condition in the context of the Rolling scene. This could be because the cybersickness intensity in this scenario was relatively low, making prior AR experience less critical for effective task engagement and, consequently, lessening its influence.

**Difference between AR cybersickness and VR cybersickness** There are researches focusing on the ”Difference between AR Cybersickness and VR Cybersickness”. Claireet al. revealed that VR users tend to experience symptoms ranging from disorientation to nausea, whereas AR users are more likely to suffer from headaches and eyestrain. Lawsont al.’s research confirmed that VR exposure typically results in more disorientation (D) than nausea (N), while AR exposure tends to cause oculomotor disturbances (O) followed by disorientation and then nausea (O>D>N profile). In light of these researches, we articulate our insights into the distinctions between cybersickness in AR as opposed to VR. We contend that the differences are twofold: immersion-induced disparities and sensory factors. VR submerges users within a fully virtual milieu, heightening immersion but concurrently obscuring their perception of the actual world. This disconnection can exacerbate sensory conflict, as users might perform actions in VR—such as climbing stairs—that do not align with their physical environment, like walking on flat ground. AR mitigates this issue by allowing users to remain visually and physically connected to their surroundings, thus tempering immersion but also reducing sensory discord.

As for the sensory factors, AR’s characteristic blend with reality suggests that visual stimuli predominantly influence the user’s experience. In contrast, VR’s entirely virtual setting can provoke cybersickness through discrepancies not just in vision but also in auditory and haptic feedback compared to the real world.

When considering the similarities of cybersickness, we propose that AR cybersickness and VR cybersickness share several key commonalities. Firstly, their triggering mechanisms are both associated with sensory conflict, where a mismatch between visual perception and other sensory systems, such as the vestibular system, can lead to the onset of cybersickness. Secondly, in terms of symptomatology, both AR and VR can induce a range of discomforts including Oculomotor symptom, Vestibular, nausea, and tension. Lastly, in terms of individual reactions, different people exhibit varying degrees of sensitivity to AR and VR cybersickness, resulting in symptoms of varying intensity. Our findings are consistent with the book “Getting Rid of Cybersickness,” which posits that VR cybersickness is more severe than AR cybersickness due to the field of view provided by HMDs and the level of immersion.

## Limitation and future works

Several limitations within this experiment merit attention. Primarily, the reliance on subjective feedback may introduce bias, as the data reflect personal experiences. To counter this, future studies will aim to incorporate objective measurements such as pupil dilation and heart rate monitoring to supplement our understanding of cybersickness^31,32^.

Moreover, our participant pool, drawn exclusively from a youthful academic setting, narrows the study’s scope. To broaden the applicability of our findings on AR-induced cybersickness, it’s crucial to include older age groups, especially given the growing use of AR among middle-aged individuals in their maintenance routines^33^. The absence of senior participants reflects an underlying assumption that AR devices are predominantly harnessed by the young and middle-aged—a demographic perceived to be more receptive to new technologies and likely to integrate them into their daily and professional lives. Moving forward, our recruitment strategy will aim to diversify our sample to better represent these varied age groups.

Another limitation is the participants’ susceptibility to cybersickness, which can reflect the likelihood of a person experiencing a cybersickness response when faced with motion-inducing situations. This can be measured using the cybersickness Susceptibility Questionnaire (MSSQ). Considering that the intensity of participants’ cybersickness could be related to their susceptibility to cybersickness, this is also one of the user characteristics that need to be considered in future research.

Lastly, the experiment’s reliance on a modified CSQ-VR questionnaire may not effectively capture high-intensity cybersickness. To address this, we introduced a tension metric, which, notably, has not consistently been a significant predictor in our findings. We speculate that the relevance of tension as a predictor may increase with cybersickness intensity, suggesting a potential area for methodological refinement in future studies. Research going forward should further examine how factors like satiety affect cybersickness, refine the thresholds discussed, and delve deeper into the effects of color and background consistency on symptoms, particularly those related to Vestibular and Oculomotor domains.

Lastly, the research conducted by Venkatakrishnan and colleagues delves into how various distractions can mitigate the effects of cybersickness, finding that auditory, cognitive, and especially visual distractions can reduce symptoms. This insight offers valuable strategies for cybersickness relief. Their study induced cybersickness through the exploration of a virtual cityscape, identifying reading as one of the potential distractions that could alleviate these effects. In contrast, our research contends that reading, while a visual distraction, may also provoke cybersickness. We suggest that reading might serve as an effective distraction not because it is symptom-free, but rather because the level of cybersickness it generates is substantially lower than that caused by navigating through a virtual environment.

## Conclusion

Our research reveals that the influence of both text display patterns and user characteristics on cybersickness is substantial. Text display elements such as intricate text movement modes, pace, and color selection are pivotal considerations for AR application developers, given their significant impact on users’ reading and searching experiences. These elements must be optimized to minimize cybersickness and safeguard user experience, particularly since cybersickness can compromise vestibular function and oculomotor control, consequently affecting decision-making and search efficiency. These insights pave the way for developing strategies to prevent and mitigate AR cybersickness.

Concurrently, the influence of user-specific traits is undeniable. Satiety, in particular, has been identified as a key predictor of cybersickness, spotlighting the need for further research to unravel the factors underpinning this effect. Moreover, past AR experience has been shown to play a pivotal role, especially as cybersickness intensifies. Thus, allowing users to acclimate to AR environments through pre-training at lower cybersickness intensities could prove beneficial, particularly in situations where exposure to higher intensity levels is inevitable.

### Supplementary Information

Supplementary Video 1.

Supplementary Legends.

## Data Availability

The datasets used and/or analyzed during the current study are available from the corresponding author upon reasonable request.
