# Supplementary material for: How different text display patterns affect cybersickness in augmented reality
Source: Sci Rep. 2024 May 22;14:11693. doi: 10.1038/s41598-024-62338-y (PMC11111777; doi:10.1038/s41598-024-62338-y)
Supplement: Supplementary file 2 — Supplementary Legends. [file 41598_2024_62338_MOESM2_ESM.pdf]

The video is a response to Reviewer 2 Comment 2, providing the demonstration of the experimental setup.
